# New Meroterpenoid Derivatives from the Pomegranate-Derived Endophytic Fungus *Talaromyces purpureogenus*

**DOI:** 10.3390/molecules28227650

**Published:** 2023-11-18

**Authors:** Alaa Anwar, Mohamed S. Elnaggar, Ahmed M. Elissawy, Nehal Ibrahim, Attila Mándi, Tibor Kurtán, Zhen Liu, Sherweit H. El-Ahmady, Rainer Kalscheuer

**Affiliations:** 1 Department of Pharmacognosy, Faculty of Pharmacy, Ain-Shams University, Abbassia, Cairo 11566, Egypt; alaa.a.husain@pharma.asu.edu.eg (A.A.); aelissawy@pharma.asu.edu.eg (A.M.E.); nehal.sabry@pharma.asu.edu.eg (N.I.); selahmady@pharma.asu.edu.eg (S.H.E.-A.); 2 Institute of Pharmaceutical Biology and Biotechnology, Heinrich Heine University, Universitätsstrasse 1, 40225 Düsseldorf, Germany; 3 Center for Drug Discovery Research and Development, Faculty of Pharmacy, Ain-Shams University, Abbassia, Cairo 11566, Egypt; 4 Department of Organic Chemistry, University of Debrecen, P.O. Box 400, 4002 Debrecen, Hungary; mandi.attila@science.unideb.hu (A.M.); kurtan.tibor@science.unideb.hu (T.K.); 5 Key Laboratory of Study and Discovery of Small Targeted Molecules of Hunan Province, School of Medicine, Hunan Normal University, Changsha 410013, China; zhenfeizi0@sina.com

**Keywords:** *Talaromyces purpureogenus*, endophytes, meroterpenoids, structural elucidation, absolute configuration, antimicrobial activity

## Abstract

In this study, we report the isolation of two new meroterpenoids, miniolutelide D (**1**) and miniolutelide E (13-*epi*-miniolutelide C) (**2**), along with two meroterpenoidal analogues (**3** and **4**) and two phenolic compounds (**5** and **6**) from the endophytic fungus *Talaromyces purpureogenus* derived from *Punica granatum* fruits. Their structures were elucidated using extensive MS, 1D, and 2D NMR spectroscopic analyses as well as by comparing with data in the literature. The absolute configurations of **1** and **2** were determined using TDDFT-ECD calculations. Antimicrobial activity was evaluated. Compound **5** displayed significant activity against methicillin-resistant *Staphylococcus aureus* strain ATCC 700699 and moderate activity against *S. aureus* strain ATCC 29213.

## 1. Introduction

Nature has granted mankind plenty of drug leads since the beginning of history. Many of these are widely used now in clinical settings and in everyday use to ameliorate various diseases [1]. Originally, plants were the major source for isolating bioactive compounds. However, endophytes have recently received much more attention from researchers due to the structural and biological diversity of their natural products. Endophytes are the micro-organisms that live within the healthy tissues of plants in a symbiotic relationship, causing no harm to their hosts [2].

Fungal endophytes represent outstanding sources of new secondary metabolites spanning different classes of secondary metabolites, including polyketides, steroids, alkaloids, peptides, terpenoids, and quinones, among others, displaying potential biological activities. Although it has been estimated that approximately one million species of endophytic fungi exist, only 5% have been investigated. There are still many fungi yet to be explored that hold many undiscovered treasures [3,4,5].

The fungus *Talaromyces purpureogenus*, formerly known as *Penicillium purpurogenum* [6], has served as a source of many secondary metabolites of biological significance, including isocoumarins, sesquiterpenoids [7], meroterpenoids [8,9], diterpenoids, alkaloids [10], steroids, phenolic derivatives [11], esters [12], anhydrides [13] and pigments [14]. These bioactive compounds showed antibacterial [7], antioxidant [12], cytotoxic [10], anti-inflammatory [13], trypanocidal [9], and anti-pseudorabies virus activities [8].

As a subclass of terpenoids, meroterpenoids are composed of isoprene units with diverse chemical structures. This high diversity is accounted for by their mixed biosynthesis, which comprises both terpenoid and polyketide pathways [15]. Various meroterpenoids have been reported to exhibit antimicrobial activity against different bacterial and fungal strains. For example, 11-hydroxychevalone E showed antibacterial activity against *Escherichia coli* and *Salmonella enterica* serovar Typhimurium. Chevalone C exhibited antibacterial activity against *Bacillus cereus* and *Staphylococcus aureus* [16]. Antifungal activity was tested on formyl phloroglucinol meroterpenoids isolated from *Eucalyptus robusta* leaves. Among them, eucalrobusone J, eucalrobusone O, and macrocarpal C showed potent antifungal activity against *Candida glabrata*, whereas eucalrobusone O and macrocarpal C showed moderate antifungal activity against *Candida albicans* [17]. Other examples worth mentioning are chrodrimanins A and B isolated from *Talaromyces funiculosus*, which exhibited both broad spectrum and some selectivity against bacterial strains, respectively. Chrodrimanin A showed broad spectrum antibacterial activity against *S. aureus*, *Mycobacterium phlei*, and *E. coli*, and was majorly selective against *Micrococcus tetragenus*. Chrodrimanin B was moderately selective against *E. coli* [18]. Significant antibacterial activity against *Helicobacter pylori* and *S. aureus* was detected in aspergillactone obtained from the marine-derived fungus *Aspergillus* sp. CSYZ-1 [19].

In the ongoing search for new bioactive compounds from natural habitats, the fungal endophyte *T. purpureogenus* isolated from the tissues of *Punica granatum* fruit was investigated. Two new meroterpenoids, miniolutelide D (**1**) and miniolutelide E (13-*epi*-miniolutelide C) (**2**), together with two known meroterpenoids, berkeleyone C (**3**) and berkeleydione (**4**) [20], and two known phenolic compounds, alternariol (**5**) [21] and 3-methylorsellinic acid (**6**) [22], were obtained. Based on the biological potentials of meroterpenoids against wide varieties of bacteria and fungi, the new meroterpenoids (**1** and **2**) identified in this study, along with known analogues (**3** and **4**), and known phenolic compounds (**5** and **6**), were screened against pathogenic bacterial strains, including *S. aureus* ATCC 29213 and methicillin-resistant *S. aureus* ATCC 700699.

## 2. Results and Discussion

**Figure 1:**
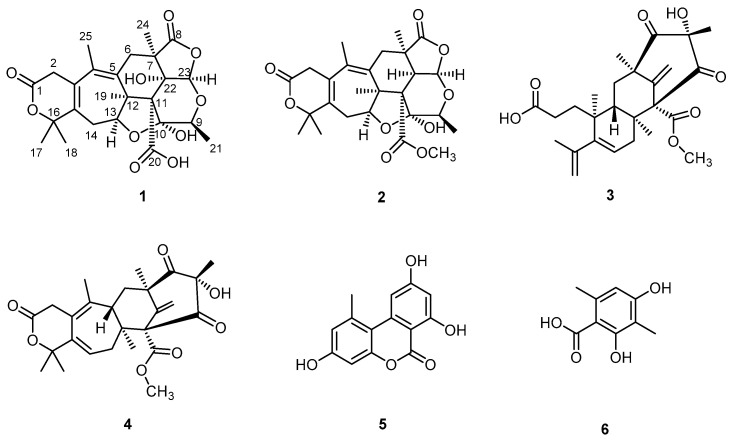
Molecular structures of isolated compounds.

Compounds (**1** and **2**) were isolated together with two known meroterpenoids, berkeleyone C (**3**) and berkeleydione (**4**) [20], and two known phenolic compounds, alternariol (**5**) [21] and 3-methylorsellinic acid (**6**) [22]. Compounds **3**–**6** were identified by comparing their obtained spectral data with those reported in the literature. The molecular structures of isolated compounds are shown in Figure 1.

Compound **1** was isolated as a white amorphous powder. HRESIMS revealed a molecular ion peak at *m*/*z* 489.1762 equivalent to C_25_H_29_O_10_ [M − H]^−^ (Figure S1). ^1^H and APT NMR data are summarized in Table 1. ^1^H NMR, APT NMR and HSQC spectra are shown in Figures S2–S4. ^1^H NMR spectrum of compound **1** (Figure S2) showed three methine protons at δ_H_ (ppm) 4.07 (1H, *q*, *J* = 6.3 Hz, H-9), 4.23 (1H, *dd*, *J* = 13, 3.75 Hz, H-13), and 5.97 (1H, *s*, H-23), where the former two methines are oxygenated, while the latter is di-oxygenated. Also, three methylene protons were found at δ_H_ (ppm) 3.34, 2.93 (2H, *d*, *J* = 21.2 Hz, H_2_-2), 2.89, 2.26 (2H, *d*, *J* = 16.2 Hz, H_2_-6), and 2.39, 2.20 (2H, *dd*, *J* = 13, 3.75 Hz; *d*, *J* = 13 Hz, H_2_-14), in addition to six methyl protons at δ_H_ (ppm) 1.42 (3H, *d*, *J* = 6.4 Hz, Me-21), 1.90 (3H, *s*, Me-25), 1.62 (3H, *s*, Me-18), 1.49 (3H, *s*, Me-17), 1.45 (3H, *s*, Me-19), and 1.38 (3H, *s*, Me-24). The APT spectrum (Figure S3) revealed the presence of 25 carbons, including 13 quaternary carbons at δ_C_ 173.36, 168.71, 164.80, 133.8, 131.65, 130.88, 128.97, 99.10, 85.05, 80.68, 72.38, 52.02, and 46.53, six methyls at δ_C_ 27.91, 27.79, 27.69, 18.64, 17.89, and 13.31, three methylenes at δ_C_ 32.51, 31.51, and 31.43, and three methine carbons at δ_C_ 98.53, 92.91, and 70.33.

By inspection of the COSY spectrum (Figure S5), it was noted that there were correlations between H-13 and H_2_-14, as well as between H-9 and H_3_-21. Additionally, the HMBC correlations (Figure S6) confirmed the planar structure of **1** where correlations of δ_H_ 3.34, 2.93 (H_2_-2) to δ_C_ 168.71 (C-1), and δ_C_ 128.97 (C-3), δ_H_ 2.89, 2.26 (H_2_-6) to δ_C_ 131.65 (C-4), δ_C_ 133.8 (C-5), δ_C_ 46.53 (C-7), δ_C_ 173.36 (C-8), δ_C_ 52.02 (C-12), δ_C_ 80.68 (C-22), and δ_C_ 18.64 (C-24), δ_H_ 4.07 (H-9) to δ_C_ 13.31 (C-21), and δ_C_ 98.53 (C-23), δ_H_ 4.23 (H-13) to δ_C_ 133.8 (C-5), and δ_C_ 27.91 (C-19), δ_H_ 2.39, 2.20 (H_2_-14) to δ_C_ 128.97 (C-3), δ_C_ 52.02 (C-12), δ_C_ 92.91 (C-13), δ_C_ 130.88 (C-15), and δ_C_ 85.05 (C-16), δ_H_ 1.49 (H_3_-17) to δ_C_ 130.88 (C-15), δ_C_ 85.05 (C-16), and δ_C_ 27.79 (C-18), δ_H_ 1.62 (H_3_-18) to δ_C_ 130.88 (C-15), δ_C_ 85.05 (C-16), and δ_C_ 27.69 (C-17), δ_H_ 1.45 (H_3_-19) to δ_C_ 133.8 (C-5), δ_C_ 72.38 (C-11), δ_C_ 52.02 (C-12), and δ_C_ 92.91 (C-13), δ_H_ 1.42 (H_3_-21) to δ_C_ 70.33 (C-9), and δ_C_ 99.10 (C-10), δ_H_ 5.97 (H-23) to δ_C_ 173.36 (C-8), and δ_C_ 72.38 (C-11), δ_H_ 1.38 (H_3_-24) to δ_C_ 32.51 (C-6), δ_C_ 46.53 (C-7), δ_C_ 173.36 (C-8), and δ_C_ 80.68 (C-22), δ_H_ 1.90 (H_3_-25) to δ_C_ 128.97 (C-3), δ_C_ 131.65 (C-4), and δ_C_ 133.8 (C-5) were observed.

From the above-mentioned data of spectroscopic analyses, it was revealed that compound **1** showed a close similarity with the previously reported data of miniolutelide C [23], showing they have a similar planar structure with only two differences. Those differences were an additional hydroxy group on the quaternary carbon C-22, accounting for the downfield shift (*δ*_c_ 80.68) compared to the methine group in miniolutelide C at (*δ*_C_ 44.80) and the absence of the proton peak at (*δ*_H_ 3.05), and also accounting for the downfield shift in C-11 from *δ*_c_ 62.1 in miniolutelide C to *δ*_c_ 72.38 in **1**. Moreover, the disappearance of the methoxy group at C-20 suggests that compound **1** is a 20-demethoxy, 22-hydroxy derivative of miniolutelide C. Compound **1** was given the trivial name miniolutelide D. Some discrepancies in ^13^C NMR data values were observed between them. C-13 found at *δ*_c_ 92.91 in **1** was located at *δ*_c_ 79.3 in miniolutelide C, in addition to C-19 found at *δ*_c_ 27.91 in **1** was at *δ*_c_ 14.9 in miniolutelide C, suggesting a possible difference in absolute configurations could be found between the two compounds.

**Figure 2:**
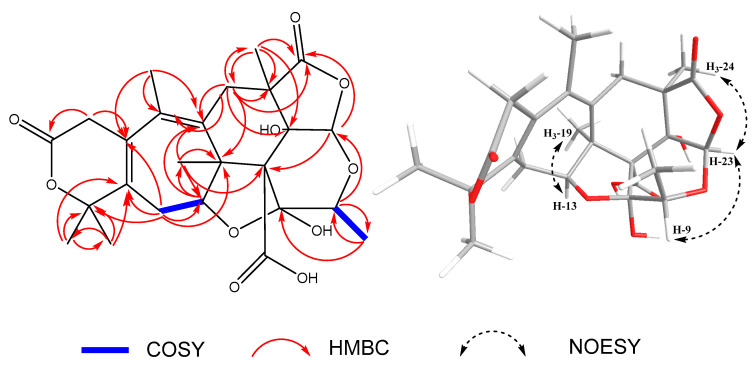
Key COSY, HMBC, and NOESY correlations of compound **1**.

The relative configuration of compound (**1**) was determined by analyzing the NOESY spectrum (Figure S7). Correlations were observed from H-23 to H_3_-24, H-9, and from H-13 to H_3_-19. Due to the characteristic NOE correlations of **1** and similarity of spectral data with those of **2**, it was assigned the same relative configuration as that of **2**; (7R*,9S*,10S*,11R*,12S*,13S*,22S*,23R*), although the descriptors of C-11 and C-22 are different due to the different priority orders. Key COSY, HMBC, and NOESY correlations are shown in Figure 2.

**Figure 3:**
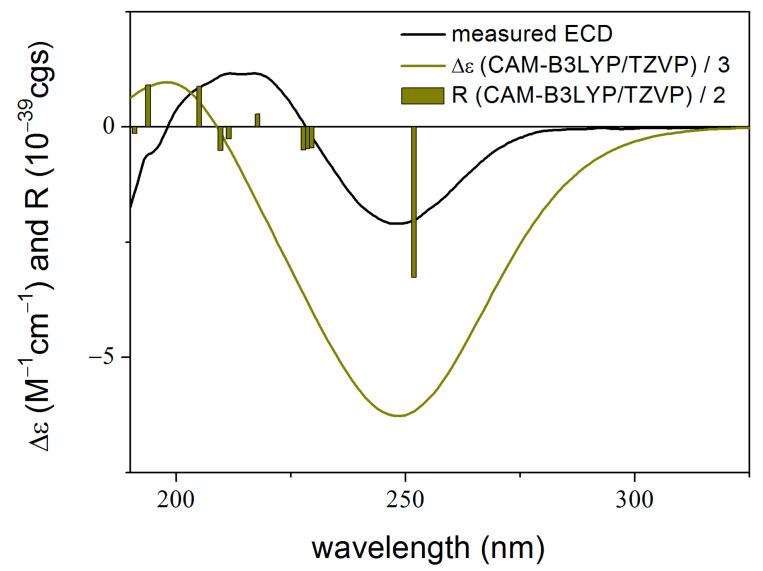
Experimental ECD spectrum of **1** measured in MeCN compared with the CAM-B3LYP/TZVP PCM/MeCN ECD spectrum of (7*R*,9*S*,10*S*,11*R*,12*S*,13*S*,22*S*,23*R*)-**1** computed for the low-energy ωB97X/TZVP PCM/MeCN conformers. The bars represent the rotational strength values of the lowest-energy conformer.

**Figure 4:**
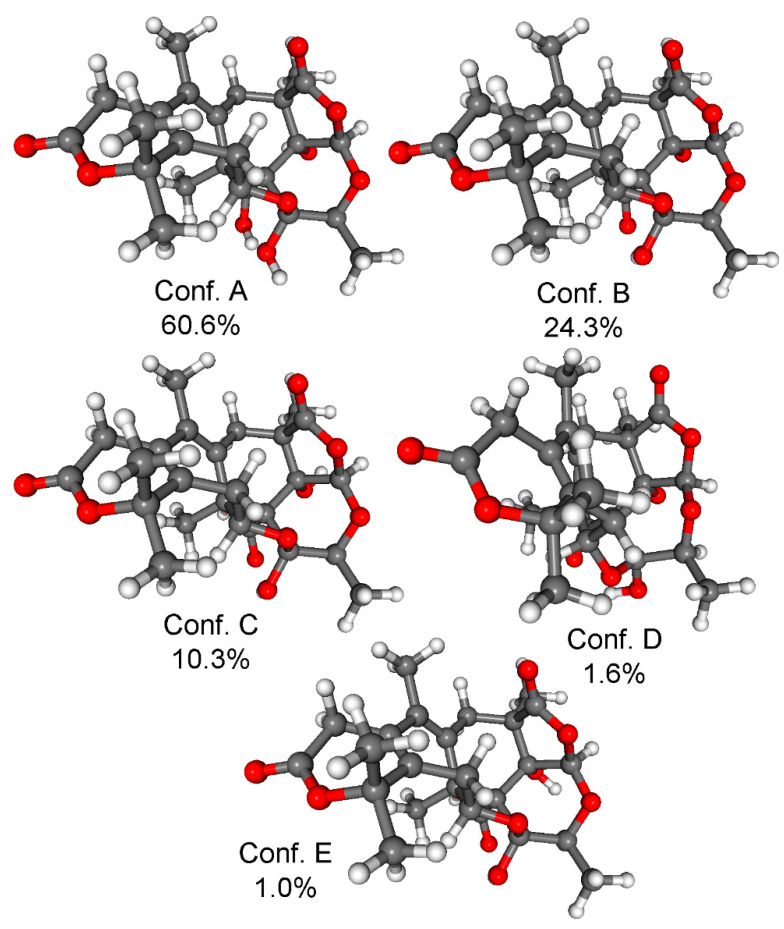
Low-energy (≥1%) conformers and Boltzmann populations of (7*R*,9*S*,10*S*,11*R*,12*S*,13*S*,22*S*,23*R*)-**1**. Level of DFT optimization: ωB97X/TZVP PCM/MeCN.

To elucidate the absolute configuration of **1**, the TDDFT-ECD method was applied to (7*R*,9*S*,10*S*,11*R*,12*S*,13*S*,22*S*,23*R*)-**1** [24,25]. The initial Merck molecular force field (MMFF) conformational search resulted in 22 conformer clusters, from which the lowest-energy ones were re-optimized at the ωB97X/TZVP PCM/MeCN level, yielding five low-energy conformers over 1% Boltzmann population. ECD spectra computed for these low-energy conformers at various levels of theory gave acceptable to good agreement with the experimental ECD spectrum. Furthermore, all low-energy conformers exhibited similar computed ECD spectra (Figure 3 and Figure 4), allowing a solid elucidation of the absolute configuration of **1** as (7*R*,9*S*,10*S*,11*R*,12*S*,13*S*,22*S*,23*R*).

Compound **2** was isolated as a yellow amorphous powder. It showed a molecular ion peak by HRESIMS at *m*/*z* 489.2114 equivalent to C_26_H_33_O_9_ [M + H]^+^ (Figure S8A,B). In Table 1 ^1^H and APT NMR data of **2** are listed. ^1^H NMR, APT NMR and HSQC spectra are shown in Figures S9–S11. ^1^H NMR spectrum of compound **2** (Figure S9) showed four methine protons at δ_H_ (ppm) 4.62 (1H, *q*, *J* = 6.5 Hz, H-9), 4.16 (1H, *dd*, *J* = 13, 4.5 Hz, H-13), 2.85 (1H, *s*, H-22), and 5.43 (1H, *s*, H-23), where the H-9 and H-13 are oxygenated, while H-23 is di-oxygenated. Three methylene protons were found at δ_H_ (ppm) 3.27, 2.83 (2H, *d*, *J* = 21.1 Hz, H_2_-2), 2.94, 2.26 (2H, *d*, *J* = 16.3 Hz, H_2_-6), and 2.66, 2.47 (2H, *t*, *J* = 13 Hz; *dd*, *J* = 13, 4.5 Hz, H_2_-14), in addition to seven methyl protons at δ_H_ (ppm) 1.24 (3H, *d*, *J* = 5.9 Hz, Me-21), 1.71 (3H, *s*, Me-25), 1.56 (3H, *s*, Me-18), 1.48 (3H, *s*, Me-17), 1.07 (3H, *s*, Me-19), 1.39 (3H, *s*, Me-24), and 3.68 (3H, *s*, Me-26). APT spectrum (Figure S10) revealed the presence of 25 carbons, including 12 quaternary carbons at δ_C_ 173.36, 169.82, 169.11, 137.35, 131.25, 129.57, 128.57, 109.34, 85.2, 57.14, 51.12, and 41.2, seven methyls at δ_C_ 51.3, 27.65, 27.47, 27.12, 21.53, 17.2, and 13.26, three methylenes at δ_C_ 42.54, 33.75, and 31.74, and four methine carbons at δ_C_ 94.47, 90.90, 67.23, and 48.78.

The COSY spectrum (Figure S12) revealed correlations between H-13 and H_2_-14, H-22 and H-23, H-9, and H_3_-21. HMBC correlations (Figure S13) confirmed the planar structure of **2,** where correlations were observed from δ_H_ 3.27, 2.83 (H_2_-2) to δ_C_ 169.11 (C-1), and δ_C_ 128.57 (C-3), δ_H_ 2.94, 2.26 (H_2_-6) to δ_C_ 129.57 (C-4), δ_C_ 137.35 (C-5), δ_C_ 41.2 (C-7), δ_C_ 173.36 (C-8), δ_C_ 51.12 (C-12), δ_C_ 48.78 (C-22), and δ_C_ 21.53 (C-24), δ_H_ 4.62 (H-9) to δ_C_ 109.34 (C-10), δ_C_ 13.26 (C-21), and δ_C_ 90.90 (C-23), δ_H_ 4.16 (H-13) to δ_C_ 137.35 (C-5), δ_C_ 109.34 (C-10), and δ_C_ 27.12 (C-19), δ_H_ 2.66, 2.47 (H_2_-14) to δ_C_ 128.57 (C-3), δ_C_ 51.12 (C-12), δ_C_ 94.47 (C-13), δ_C_ 131.25 (C-15), and δ_C_ 85.2 (C-16), δ_H_ 1.48 (H_3_-17) to δ_C_ 131.25 (C-15), δ_C_ 85.2 (C-16), and δ_C_ 27.47 (C-18), δ_H_ 1.56 (H_3_-18) to δ_C_ 131.25 (C-15), δ_C_ 85.2 (C-16), and δ_C_ 27.65 (C-17), δ_H_ 1.07 (H_3_-19) to δ_C_ 137.35 (C-5), δ_C_ 57.14 (C-11), δ_C_ 51.12 (C-12), and δ_C_ 94.47 (C-13), δ_H_ 1.24 (H_3_-21) to δ_C_ 67.23 (C-9), and δ_C_ 109.34 (C-10), δ_H_ 2.85 (H-22) to δ_C_ 41.2 (C-7), δ_C_ 173.36 (C-8), δ_C_ 109.34 (C-10), δ_C_ 57.14 (C-11), δ_C_ 90.90 (C-23), and δ_C_ 21.53 (C-24), δ_H_ 5.43 (H-23) to δ_C_ 41.2 (C-7), δ_C_ 67.23 (C-9), δ_C_ 57.14 (C-11), and δ_C_ 48.78 (C-22), δ_H_ 1.39 (H_3_-24) to δ_C_ 42.54 (C-6), δ_C_ 41.2 (C-7), δ_C_ 173.36 (C-8), and δ_C_ 48.78 (C-22), δ_H_ 1.71 (H_3_-25) to δ_C_ 128.57 (C-3), δ_C_ 129.57 (C-4), and δ_C_ 137.35 (C-5), δ_H_ 3.68 (H_3_-26) to δ_C_ 169.82 (C-20).

Hence, the planar structure of **2** was found to be the same as that of miniolutelide C [23]. However, C-13 was found at *δ*_c_ 94.47 in **2**, while it was located at *δ*_c_ 79.3 in miniolutelide C. The large difference between the chemical shift values suggests that the relative configuration might be different.

**Figure 5:**
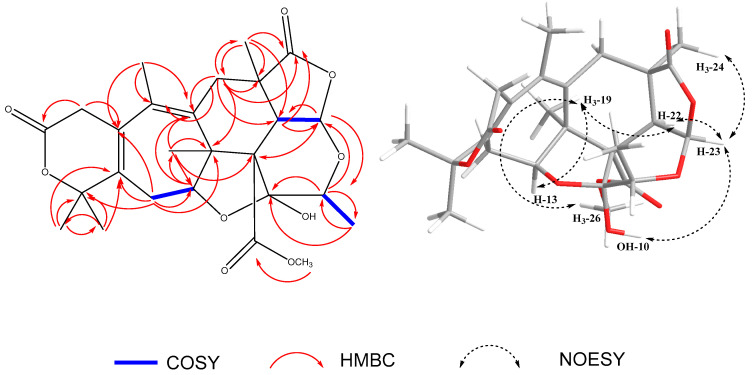
Key COSY, HMBC, and NOESY correlations of compound **2**.

After being measured in CDCl_3_ (Figures S9 and S14), the ^1^H NMR and NOESY spectra were measured in dimethylsulfoxide (DMSO) (Figures S15 and S16) to confirm the relative configuration of **2**. NOE correlations were observed from H-22 to H_3_-24, H_3_-19, and H-23, from H-23 to H_3_-24, from H_3_-19 to H-13, and H_3_-26, from OH-10 to H-23, and H-9, indicating that they are on the same side. Since H-9 is on the prior plane and H-21 showed no correlations with any of that plane’s protons, this suggests that H-21 is on the opposite face. By comparing the relative configuration of **2** with that of miniolutelide C [23], a difference in C-13 was observed, where its configuration is (S*) instead of (R*). Therefore, the relative configuration was determined as (7R*,9S*,10S*,11S*,12S*,13S*,22R*,23R*). Thus, **2** is a new metabolite for which the trivial name miniolutelide E (13*-epi*-miniolutelide C) was given. Key COSY, HMBC, and NOESY correlations are shown in Figure 5.

**Figure 6:**
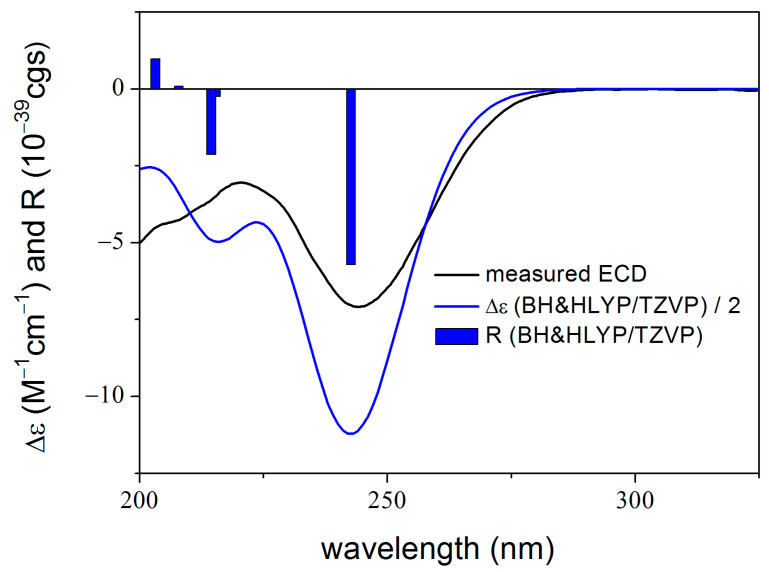
Experimental ECD spectrum of **2** measured in MeCN compared with the BH&HLYP/TZVP PCM/MeCN ECD spectrum of (7*R*,9*S*,10*S*,11*S*,12*S*,13*S*,22*R*,23*R*)-**2** computed for the single major ωB97X/TZVP PCM/MeCN conformer. The bars represent the rotational strength values of the lowest-energy conformer.

**Figure 7:**
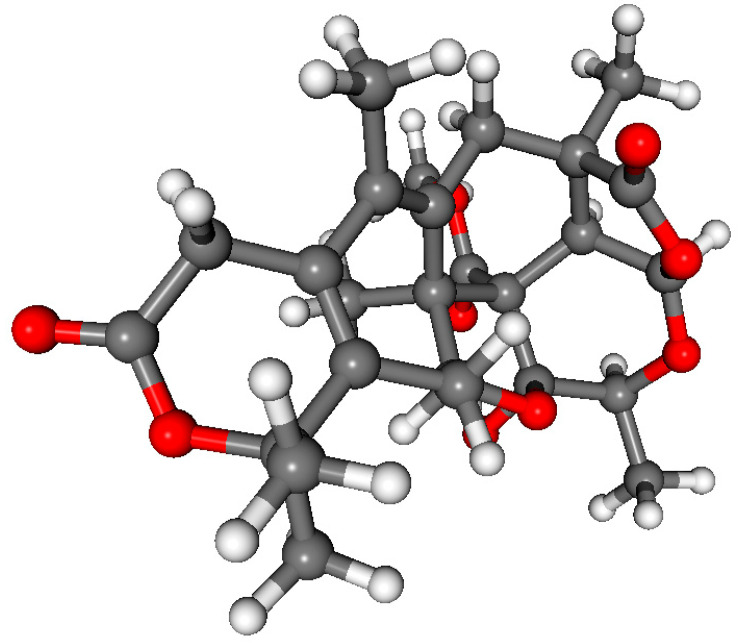
The single computed conformer of (7*R*,9*S*,10*S*,11*S*,12*S*,13*S*,22*R*,23*R*)-**2** with a Boltzmann population of 99.3% optimized at the ωB97X/TZVP PCM/MeCN level.

The absolute configuration of **2** was also confirmed by TDDFT-ECD calculations computing for (7*R*,9*S*,10*S*,11*S*,12*S*,13*S*,22*R*,23*R*)-**2**. DFT re-optimization of the initial 9 MMFF conformers resulted in a single major conformer with 99.3% Boltzmann population. ECD spectra computed at various levels for this conformer gave acceptable to good agreement with the experimental ECD spectrum (Figure 6 and Figure 7), allowing determination of the absolute configuration as (7*R*,9*S*,10*S*,11*S*,12*S*,13*S*,22*R*,23*R*)-**2**.

The emergence and prevalence of antimicrobial resistance are currently depicted as a worldwide public health threat. Most pathogens develop resistance to common antimicrobials, which risk treatment regimens’ failure. Therefore, the urgent need for new antimicrobial agents to combat this serious problem is steadily increasing [26,27]. Inhibitory activity of the total crude extract was evaluated against the pathogenic gram-positive bacterial strain *S. aureus* ATCC 29213, which causes food poisoning, toxic shock syndrome, and scalded skin syndrome, among other diseases, as well as the Gram-negative bacterial strain *E. coli* ATCC 25922 known to cause food-borne illnesses [28]. The extract was only significantly active against *S. aureus* ATCC 29213, with an MIC value of 6.3 µg/mL. Subsequently, all isolated compounds **1**–**6** were tested for antimicrobial activity against *S. aureus* ATCC 29213, as well as the methicillin-resistant *S. aureus* (MRSA) strain ATCC 700699. Only compound **5** exhibited considerable activity against *S. aureus* ATCC 700699 with an MIC of 25 µM, and modest activity against *S. aureus* ATCC 29213 with an MIC of 100 µM. None of the other compounds showed significant activity at a dose of 100 µM.

Tuberculosis, an infectious disease caused by *M. tuberculosis*, is the major cause of death by an infectious bacterium, accounting for more than a million deaths every year. Although huge efforts are being made by health communities, the morbidity and mortality rates are still high, especially in developing countries, along with the problem of emerging resistant strains, which put great importance in finding new anti-tubercular agents [29,30]. Hence, the total extract as well as all isolated compounds were tested against *M. tuberculosis*. However, none of them showed any significant activity at 100 µM.

## 3. Materials and Methods

### 3.1. General Experimental Procedures

NMR spectra were recorded on a Bruker Avance III HD 400 MHz NMR spectrometer (Zurich, Switzerland). LC-MS Shimadzu 8045 spectrometer coupled with photodiode array (PDA) detector (LC-2030/2040) was used to record the mass spectra. HRESIMS spectra were acquired on FTHRMS-Orbitrap (Thermo-Finnigan, Weiler bei Bingen, Germany) mass spectrometer. Semi-preparative HPLC (Shimadzu, Kyoto, Japan) was utilized for purification, with Kromasil C-18 RP semi-preparative column (10 mm × 250 mm), with 5 mL/min flow rate, and UV detection at 254 nm with λ max absorption at 220–400 nm. Medium-pressure liquid chromatography was performed using the Puriflash 4125 system, Montlucon, France (Interchim software, Intersoft v5.0b09) coupled with a PDA detector. HiMedia silica gel GRM7484 (0.037–0.063 mm) was utilized for column chromatography. TLC analyses were carried out on precoated silica gel 60 F_254_ plates (Merck, Darmstadt, Germany), followed by UV detection at 254 and 365 nm and spraying with vanillin sulphuric acid reagent. All solvents used were priorly distilled, and spectroscopic grade solvents were used for spectroscopic measurements.

### 3.2. Fungal Material and Identification

Pomegranate fruit collected in Cairo, Egypt, in the summer of 2019 was identified and authenticated as *P. granatum*, family Lythraceae. The fruit was cleaned and cut longitudinally. Inside the aseptic area, different fruit parts were cut by sterile blades, washed with distilled water, followed by treatment with 70% ethanol to exclude any contaminants and epiphytes that might arise. The sterile dissected parts were inoculated aseptically on malt extract agar medium (MEA). Plates were grown at room temperature (25 °C). After successive purification steps, the pure fungal strain was successfully isolated from the tissues of the *P. granatum* fruit. The same fungal strain was simultaneously isolated from the mesocarp, calyx, and exocarp, which confirms its indigeneity in *P. granatum* fruits. The fungus was identified as *T. purpureogenus* according to a molecular biological protocol using DNA amplification and sequencing of the ITS region [31]. The obtained sequencing data were submitted to Genbank with accession number OM367903. A voucher plate for this fungal strain was deposited in the Department of Pharmacognosy, Faculty of Pharmacy, Ain-Shams University, with the ID code AMP-R-2.

### 3.3. Fermentation, Fractionation, and Isolation of Bioactive Compounds

Solid rice medium was prepared in 22 1-L Erlenmeyer flasks, each containing 100 g of commercial rice and 110 mL of water. The solid rice medium was autoclaved at 121 °C for 20 min prior to fungal fermentation. The fungal strain was cultivated on the rice medium for 7 weeks at room temperature. Afterwards, fungal biomass was extracted using 700 mL ethyl acetate (EtOAc) for each flask. The flasks were left in static mode overnight. The following day, the flasks were put on electric shakers at 150 rpm for 8 h. Filtration and evaporation steps were followed to obtain a dried extract. The extraction step was run four successive times, yielding 75 g of crude extract.

The crude extract was partitioned between *n*-hexane and 90% aqueous methanol (aq. MeOH) yielding 40 g of methanolic extract. The obtained methanolic extract was fractionated by vacuum liquid chromatography (VLC) with silica gel 230–400 mesh-packed column. Gradient elution was performed using *n*-hexane–EtOAc 100:0 to 0:100 and CH_2_Cl_2_–MeOH 100:0 to 0:100, collecting 23 fractions (F1–F23). All fractions were evaporated and weighed.

Fraction F4 (2.14 g), eluted from *n*-hexane–EtOAc 1:1, was subjected to further chromatographic fractionation by VLC (230–400 mesh), applying the same described technique. This resulted in 12 sub-fractions (F4-1 to F4-12). Sub-fractions F4-5 and F4-6, obtained from 60% and 40% *n*-hexane, respectively, were combined together based on high similarity in their thin layer chromatography (TLC) pattern and thus named F-4-6c (850 mg). Semi-preparative HPLC was performed on sub-fraction F-4-6c, which led to the isolation of four compounds, **1** (9.2 mg), **3** (17.2 mg), **4** (20.1 mg), and **5** (10.4 mg).

Fractions 7, 8, and 9 eluted from *n*-hexane-EtOAc 8:2, 9:1 and 10:0, respectively, were combined (2.0 g), and subjected to another VLC subfractionation, from which 27 sub-fractions were obtained. Sub-fractions collected from *n*-hexane-EtOAc 4:6 and 3:7 (twice) F7-9, F7-10, and F7-11 were combined obtaining “F7-9+”, which was subjected to flash chromatography yielding compound **2** (20.0 mg).

While running the VLC on the total extract, an orange band F-3′ was observed in a 60% *n*-hexane solvent system, which was collected separately from the original fraction F-3. F-3′ (590 mg) was subjected to semi-preparative HPLC to yield **6** (7.4 mg).

All fractionation and isolation steps were continually tracked by TLCs of isolated fractions, sub-fractions, and compounds in different mobile phase systems.

### 3.4. Antimicrobial Susceptibility Tests

The broth micro-dilution method was applied to evaluate the antimicrobial activity against *Staphylococcus aureus* ATCC 29213, *Staphylococcus aureus* ATCC 700699, *Escherichia coli* ATCC 25922, and the minimum inhibitory concentration (MIC) values were measured according to the Clinical and Laboratory Standards Institute (CLSI) recommendations [32]. The MIC against *Mycobacterium tuberculosis* H37Rv was determined in 96-well microtiter plates employing the resazurin reduction assay, as described previously [33]. Moxifloxacin and ciprofloxacin were used as positive controls for *S. aureus* and *E. coli*, whereas rifampicin was used as a positive control for *M. tuberculosis*. DMSO was used as the solvent control.

### 3.5. Computational Section

Mixed torsional/low-frequency mode conformational searches were carried out by means of the Macromodel 10.8.011 software using the MMFF with an implicit solvent model for CHCl_3_ [34]. Geometry re-optimizations were carried out at the ωB97X/TZVP level [35] with the PCM solvent model for MeCN. TDDFT-ECD calculations were run with various functionals (B3LYP, BH&HLYP, CAMB3LYP, and PBE0) and the TZVP basis set as implemented in the Gaussian 09 package with the same solvent model as in the preceding DFT optimization step [36]. ECD spectra were generated as sums of Gaussians with 2700 and 4200 cm^−1^ width at half-height, using dipole-velocity-computed rotational strength values [37]. Boltzmann distributions were estimated from the ωB97X energies. The Molekel software package (v5.4) was used for the visualization of the results [38].

## 4. Conclusions

Chemical investigation of the endophytic fungus *Talaromyces purpureogenus*, derived from *Punica granatum* fruits, led to the isolation of two new meroterpenoids together with two other known meroterpenoids and two known phenolic compounds. The compounds were identified based on their spectral data and by comparison with previously reported data in the literature. The absolute configuration of newly isolated compounds was confirmed by TDDFT-ECD calculations. Additionally, antimicrobial activity was evaluated. Compound **5** displayed significant activity against the MRSA strain *S. aureus* ATCC 700699, as well as moderate activity against the drug-susceptible reference strain *S. aureus* ATCC 29213. Further biological investigations of the isolated compounds from *T. purpureogenus* are suggested for future research.

## Figures and Tables

**Table 1:** ^1^H and APT NMR data for compounds **1** and **2** in CDCl_3_.

Position	*δ*_C_ 1	*δ*_H_1, Multiplicity (*J* in Hz)	*δ*_C_ 2	*δ*_H_2, Multiplicity (*J* in Hz)
1	168.71, C		169.11, C	
2	31.51, CH_2_	β 3.34, d (21.2)	31.74, CH_2_	β 3.27, d (21.1)
		α 2.93, d (21.2)		α 2.83, d (21.1)
3	128.97, C		128.57, C	
4	131.65, C		129.57, C	
5	133.8, C		137.35, C	
6	32.51, CH_2_	β 2.89, d (16.2)	42.54, CH_2_	β 2.94, d (16.3)
		α 2.26, d (16.2)		α 2.26, d (16.3)
7	46.53, C		41.2, C	
8	173.36, C		173.36, C	
9	70.33, CH	4.07, q (6.3)	67.23, CH	4.62, q (6.5)
10	99.10, C		109.34, C	
11	72.38, C		57.14, C	
12	52.02, C		51.12, C	
13	92.91, CH	4.23, dd (13, 3.75)	94.47, CH	4.16, dd (13, 4.5)
14	31.43, CH_2_	β 2.39, dd (13, 3.75)	33.75, CH_2_	β 2.66, t (13)
		α 2.20, d (13)		α 2.47, dd (13, 4.5)
15	130.88, C		131.25, C	
16	85.05, C		85.2, C	
17	27.69, CH_3_	1.49, s	27.65, CH_3_	1.48, s
18	27.79, CH_3_	1.62, s	27.47, CH_3_	1.56, s
19	27.91, CH_3_	1.45, s	27.12, CH_3_	1.07, s
20	164.80, C		169.82, C	
21	13.31, CH_3_	1.42, d (6.4)	13.26, CH_3_	1.24, d (5.9)
22	80.68, C		48.78, CH	2.85, s
23	98.53, CH	5.97, s	90.90, CH	5.43, s
24	18.64, CH_3_	1.38, s	21.53, CH_3_	1.39, s
25	17.89, CH_3_	1.90, s	17.2, CH_3_	1.71, s
26			51.3, CH_3_	3.68, s

## Data Availability

Data generated and/or analyzed in this study are available in the manuscript or the Supplementary Materials or can be requested from the corresponding authors.

## Supplementary Materials

The following supporting information can be downloaded at https://www.mdpi.com/article/10.3390/molecules28227650/s1: Figures S1–S7: ESI-MS, ^1^H NMR, APT NMR, HSQC, COSY, HMBC, and NOESY spectra for compound **1**; Figures S8–S16: ESI-MS, ^1^H NMR, APT NMR, HSQC, COSY, HMBC, and NOESY spectra for compound **2**.
