# Supplementary material for: New Meroterpenoid Derivatives from the Pomegranate-Derived Endophytic Fungus Talaromyces purpureogenus
Source: Molecules. 2023 Nov 18;28(22):7650. doi: 10.3390/molecules28227650 (PMC10673506; doi:10.3390/molecules28227650)
Supplement: Supplementary file 1 [file molecules-28-07650-s001.zip › molecules-2696654-supplementary.pdf]

Supporting Information

New Meroterpenoid Derivatives from the Pomegranate-Derived Endophytic Fungus *Talaromyces purpureogenus*

Alaa Anwar [1], Mohamed S. Elnaggar [1,2,*], Ahmed M. Elissawy [1,3], Nehal Ibrahim [1], Attila Mándi [4], Tibor Kurtán [4], Zhen Liu [5], Sherweit H. El-Ahmady [1] and Rainer Kalscheuer [2,*]

[1] Department of Pharmacognosy, Faculty of Pharmacy, Ain-Shams University, Abbassia, Cairo 11566, Egypt; alaa.a.husain@pharma.asu.edu.eg (A.A.); aelissawy@pharma.asu.edu.eg (A.M.E.); nehal.sabry@pharma.asu.edu.eg (N.I.); selahmady@pharma.asu.edu.eg (S.H.E.-A.)

[2] Institute of Pharmaceutical Biology and Biotechnology, Heinrich Heine University, Universitätsstrasse 1, 40225 Düsseldorf, Germany

[3] Center for Drug Discovery Research and Development, Faculty of Pharmacy, Ain-Shams University, Abbassia, Cairo 11566, Egypt

[4] Department of Organic Chemistry, University of Debrecen, P.O. Box 400, 4002 Debrecen, Hungary; mandi.attila@science.unideb.hu (A.M.); kurtan.tibor@science.unideb.hu (T.K.)

[5] Key Laboratory of Study and Discovery of Small Targeted Molecules of Hunan Province, School of Medicine, Hunan Normal University, Changsha 410013, China; zhenfeizi0@sina.com

* Correspondence: mohamed.s.elnaggar@pharma.asu.edu.eg (M.S.E.); rainer.kalscheuer@uni-duesseldorf.de (R.K.); Tel.: +20-1005647840 (M.S.E.); +49-211-81-14180 (R.K.)

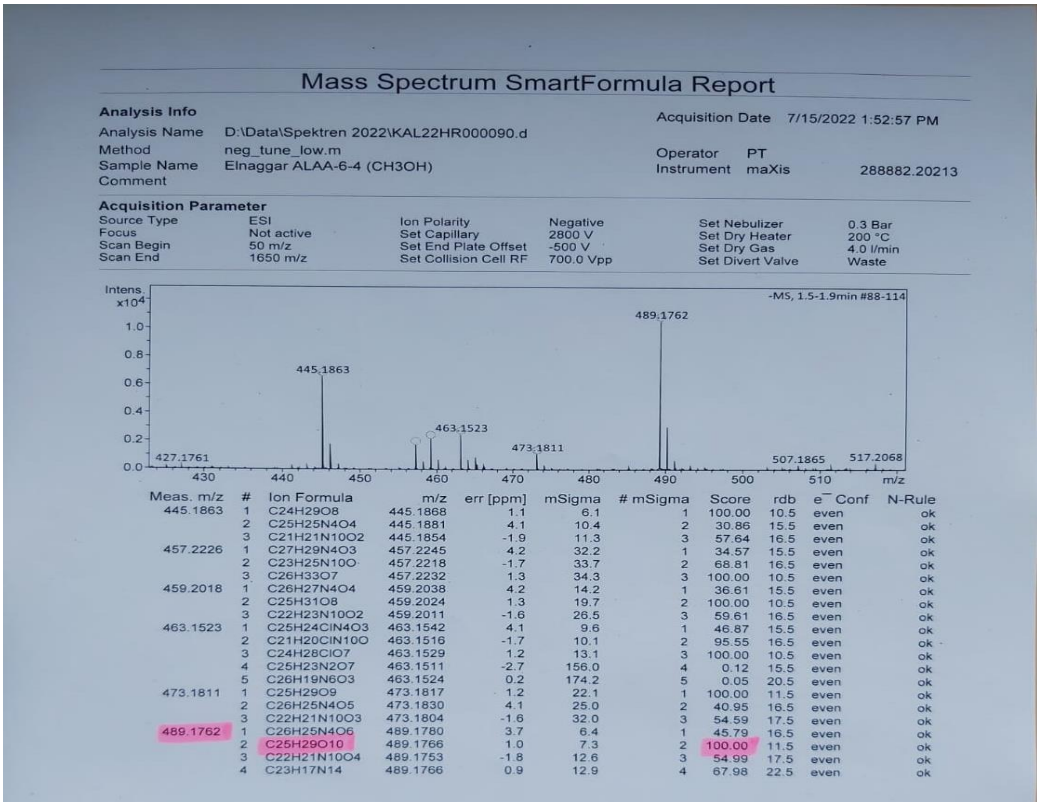

Mass Spectrum SmartFormula Report

Analysis Info

Analysis Name D:\Data\Spektren 2022\KAL22HR000090.d
Method neg_tune_low.m
Sample Name Elnaggar ALAA-6-4 (CH3OH)
Comment

Acquisition Date 7/15/2022 1:52:57 PM
Operator PT
Instrument maXis 288882.20213

Acquisition Parameter

Source Type	ESI	Ion Polarity	Negative	Set Nebulizer	0.3 Bar
Focus	Not active	Set Capillary	2800 V	Set Dry Heater	200 °C
Scan Begin	50 m/z	Set End Plate Offset	-500 V	Set Dry Gas	4.0 l/min
Scan End	1650 m/z	Set Collision Cell RF	700.0 Vpp	Set Divert Valve	Waste

Meas. m/z	#	Ion Formula	m/z	err [ppm]	mSigma	# mSigma	Score	rdb	e⁻ Conf	N-Rule
445.1863	1	C24H29O8	445.1868	1.1	6.1	1	100.00	10.5	even	ok
	2	C25H25N4O4	445.1881	4.1	10.4	2	30.86	15.5	even	ok
	3	C21H21N10O2	445.1854	-1.9	11.3	3	57.64	16.5	even	ok
457.2226	1	C27H29N4O3	457.2245	4.2	32.2	1	34.57	15.5	even	ok
	2	C23H25N10O	457.2218	-1.7	33.7	2	68.81	16.5	even	ok
	3	C26H33O7	457.2232	1.3	34.3	3	100.00	10.5	even	ok
459.2018	1	C26H27N4O4	459.2038	4.2	14.2	1	36.61	15.5	even	ok
	2	C25H31O8	459.2024	1.3	19.7	2	100.00	10.5	even	ok
	3	C22H23N10O2	459.2011	-1.6	26.5	3	59.61	16.5	even	ok
463.1523	1	C25H24ClN4O3	463.1542	4.1	9.6	1	46.87	15.5	even	ok
	2	C21H20ClN10O	463.1516	-1.7	10.1	2	95.55	16.5	even	ok
	3	C24H28ClO7	463.1529	1.2	13.1	3	100.00	10.5	even	ok
	4	C25H23N2O7	463.1511	-2.7	156.0	4	0.12	15.5	even	ok
	5	C26H19N6O3	463.1524	0.2	174.2	5	0.05	20.5	even	ok
473.1811	1	C25H29O9	473.1817	1.2	22.1	1	100.00	11.5	even	ok
	2	C26H25N4O5	473.1830	4.1	25.0	2	40.95	16.5	even	ok
	3	C22H21N10O3	473.1804	-1.6	32.0	3	54.59	17.5	even	ok
489.1762	1	C26H25N4O6	489.1780	3.7	6.4	1	45.79	16.5	even	ok
	2	C25H29O10	489.1766	1.0	7.3	2	100.00	11.5	even	ok
	3	C22H21N10O4	489.1753	-1.8	12.6	3	54.99	17.5	even	ok
	4	C23H17N14	489.1766	0.9	12.9	4	67.98	22.5	even	ok

Figure S1. High resolution –ve ESIMS of compound **1**

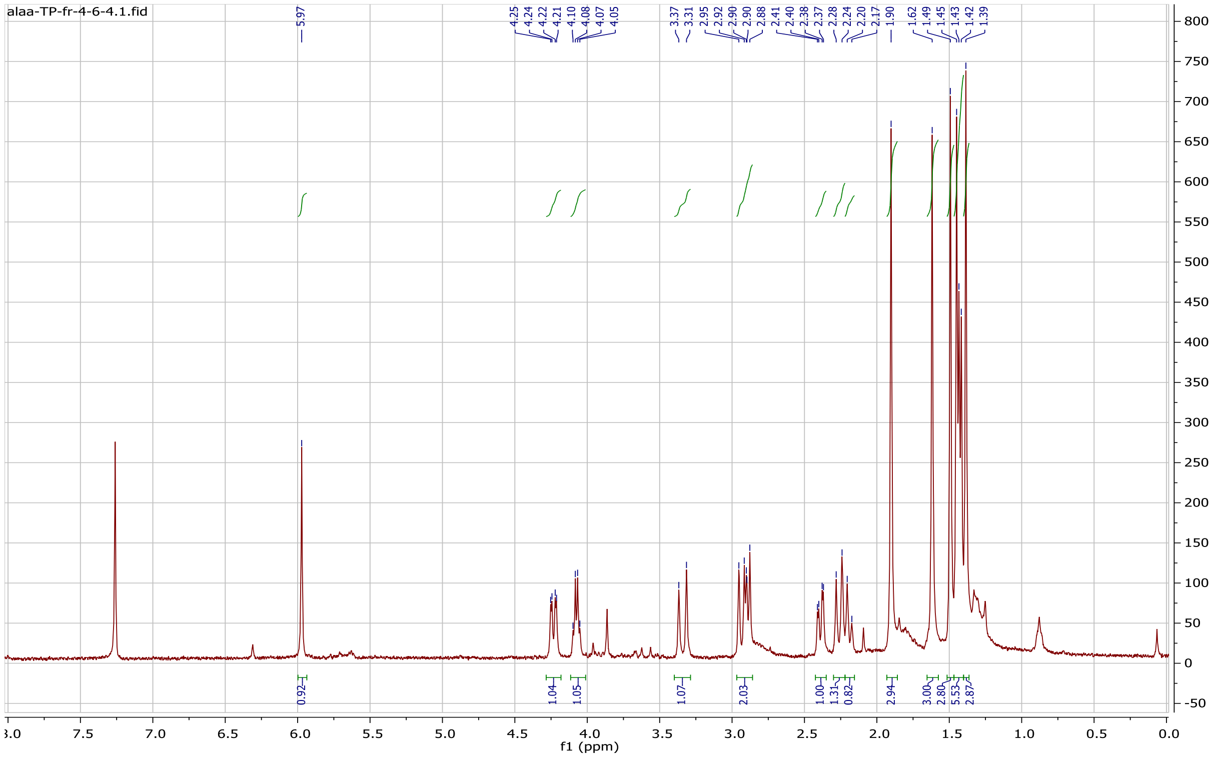

Figure S2. ^{1}H NMR spectrum of compound **1** in $CDCl_3$

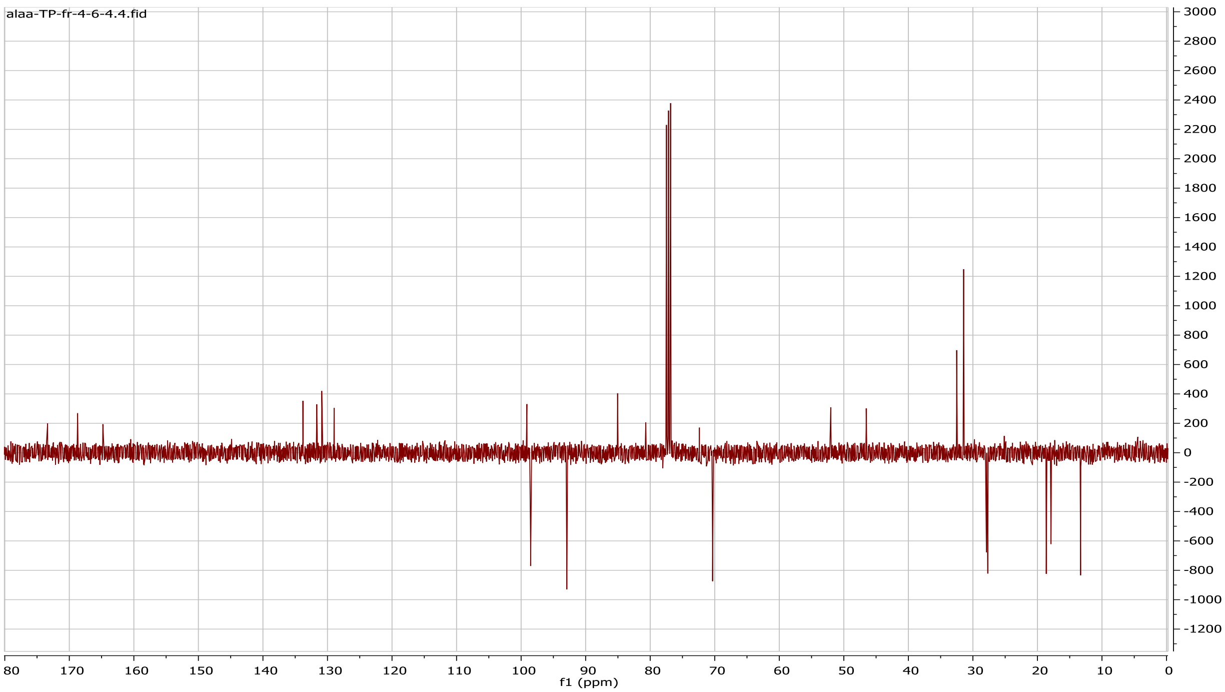

Figure S3. APT NMR spectrum of compound **1** in $CDCl_3$

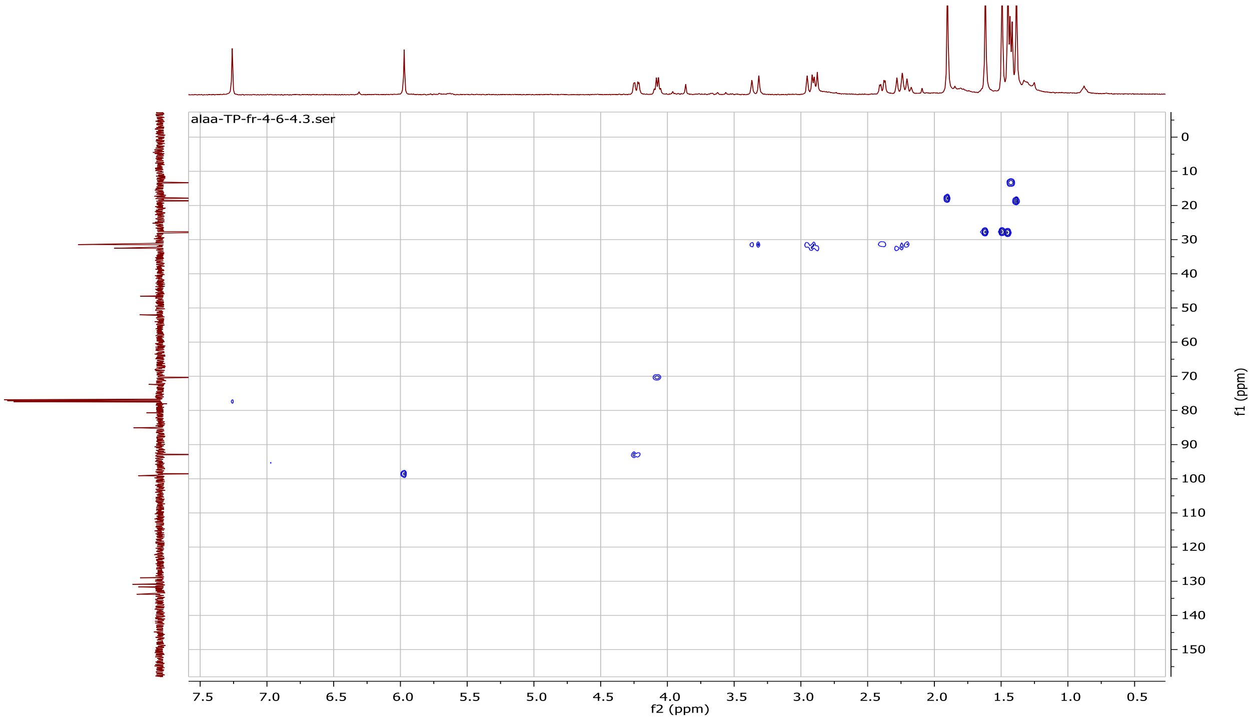

Figure S4. HSQC spectrum of compound **1** in $CDCl_3$

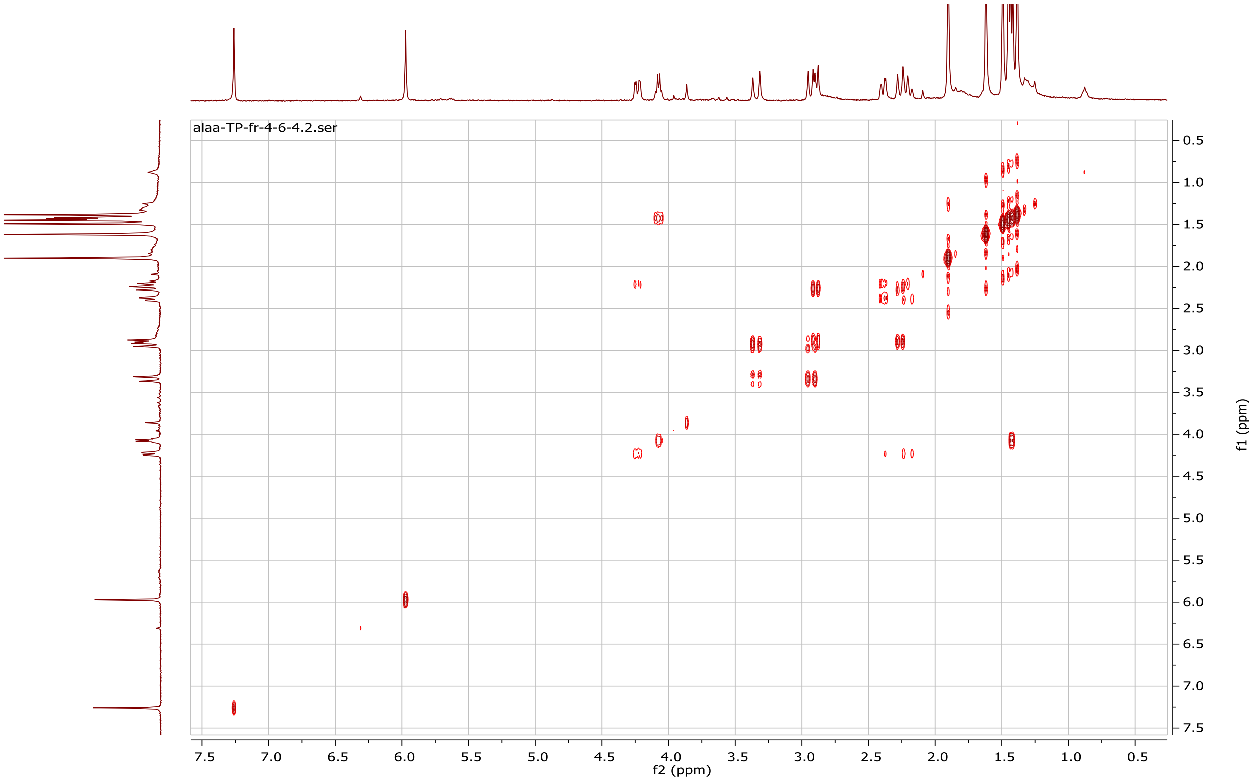

Figure S5. COSY spectrum of compound **1** in $CDCl_3$

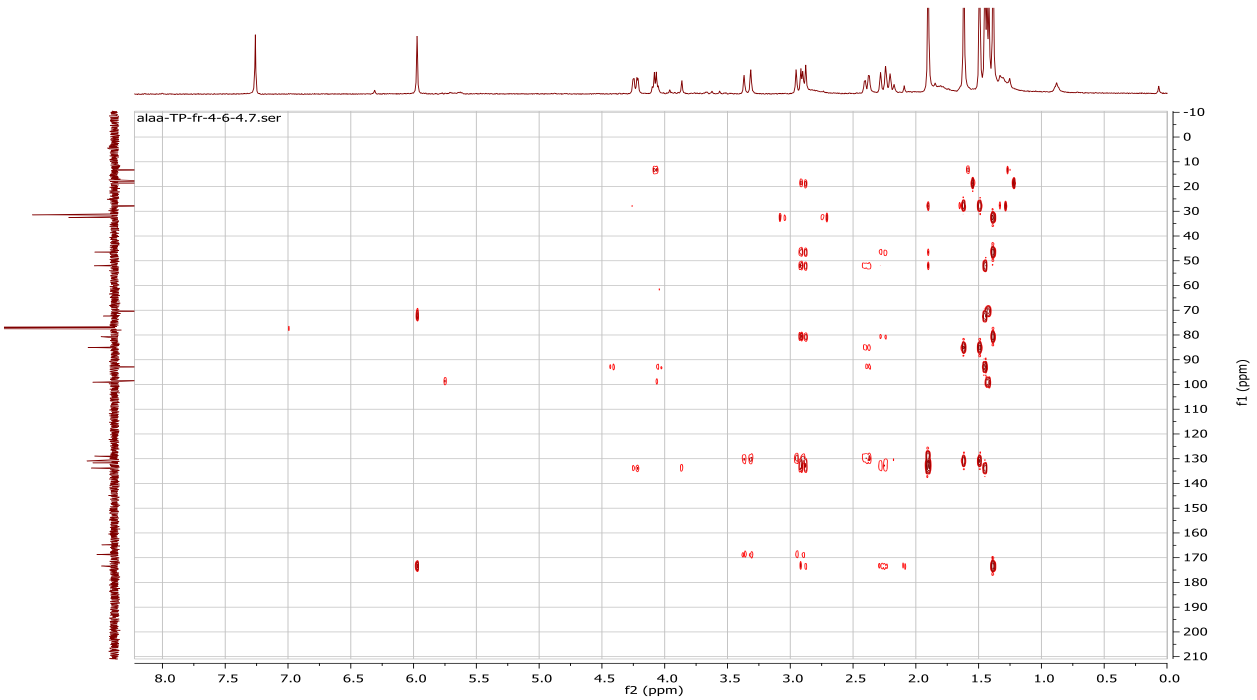

Figure S6. HMBC spectrum of compound **1** in $CDCl_3$

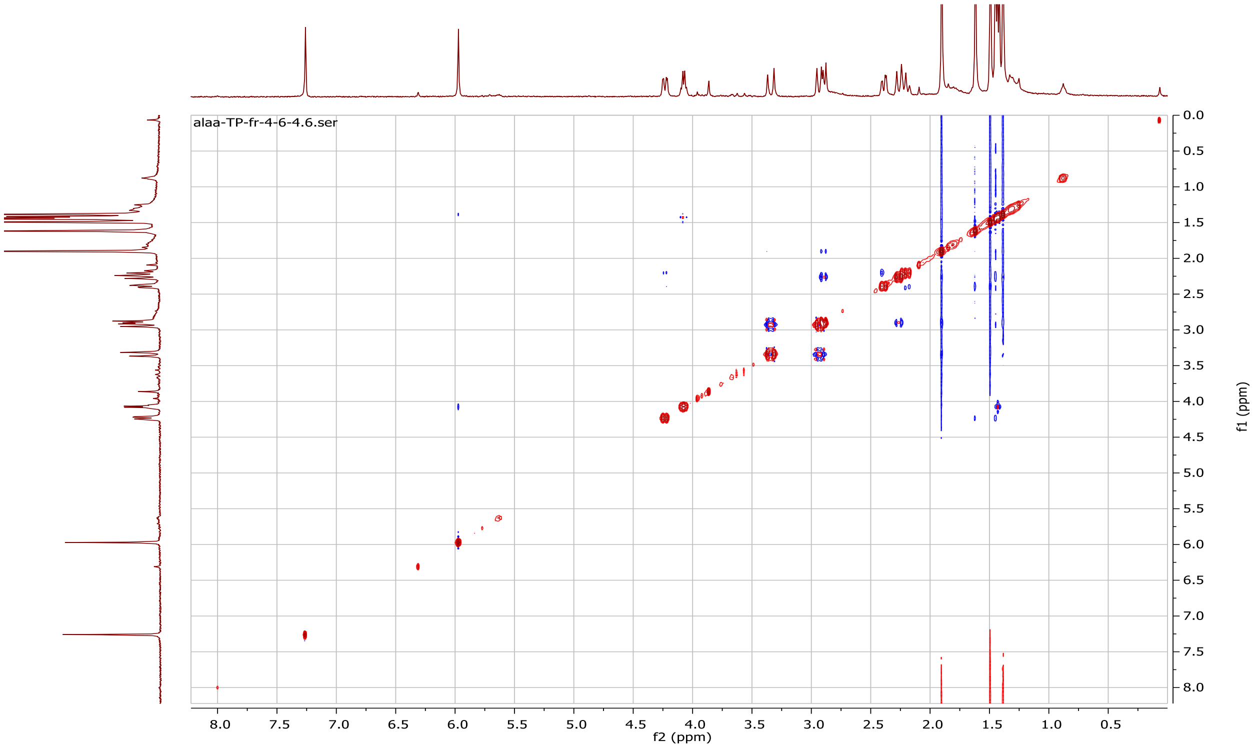

Figure S7. NOESY spectrum of compound **1** in $CDCl_3$

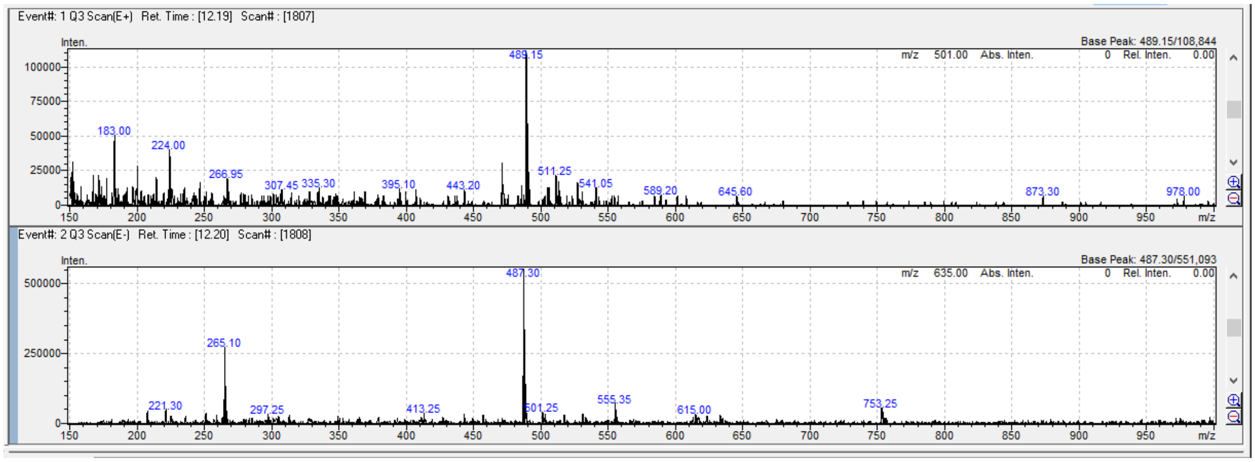

(**A**)

Mass Spectrum SmartFormula Report

Analysis Info

Analysis Name	D:\Data\Spektren 2022\KAL22HR000143.d	Acquisition Date	10/24/2022 3:33:03 PM
Method	tune_low_new.m	Operator	PT
Sample Name	Elnaggar TP-7-9-9 (CH3OH)	Instrument	maXis 288882.20213
Comment			

Acquisition Parameter

Source Type	ESI	Ion Polarity	Positive	Set Nebulizer	0.3 Bar
Focus	Not active	Set Capillary	4000 V	Set Dry Heater	180 °C
Scan Begin	50 m/z	Set End Plate Offset	-500 V	Set Dry Gas	4.0 l/min
Scan End	1500 m/z	Set Collision Cell RF	600.0 Vpp	Set Divert Valve	Source

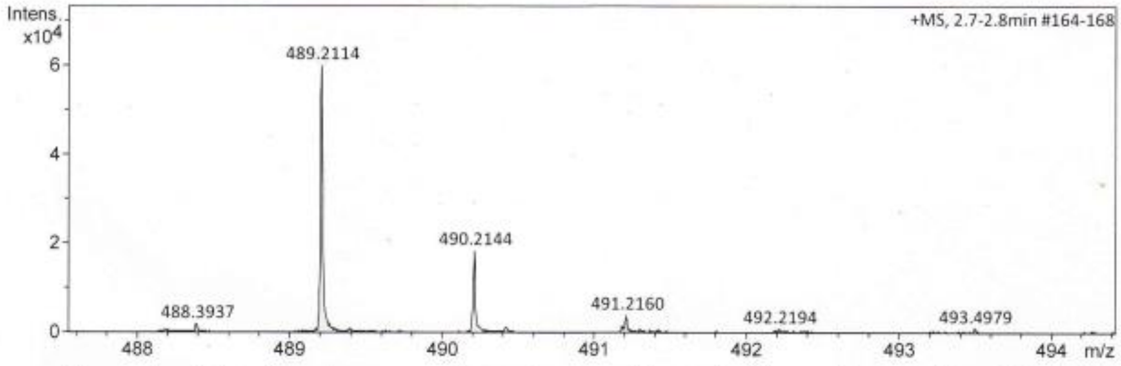

Meas. m/z	#	Ion Formula	m/z	err [ppm]	mSigma	# mSigma	Score	rdb	e⁻ Conf	N-Rule
489.2114	1	C26H33O9	489.2119	1.0	10.7	1	100.00	10.5	even	ok
	2	C23H25N10O3	489.2106	-1.7	15.0	2	57.31	16.5	even	ok

(**B**)

Figure S8. (**A**) Low resolution +ve/–ve ESIMS of compound **2**. (**B**) High resolution +ve ESIMS of compound **2**.

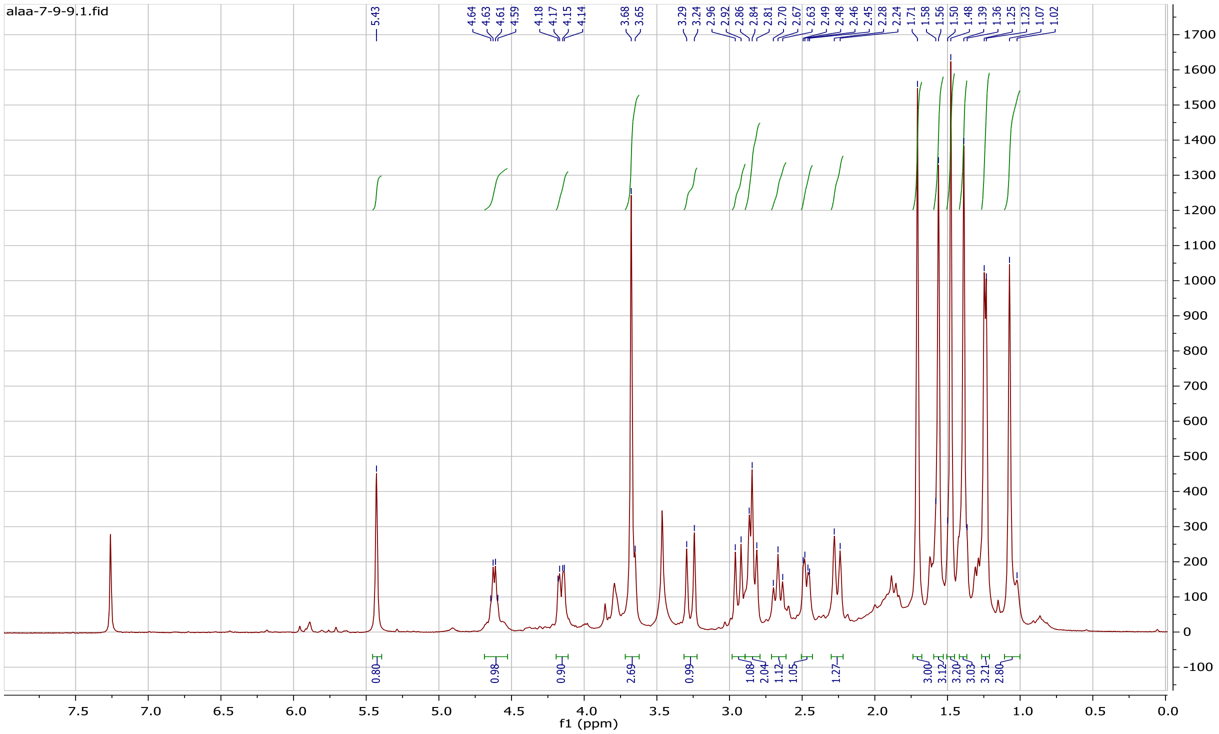

Figure S9. ^1H NMR spectrum of compound **2** in $CDCl_3$

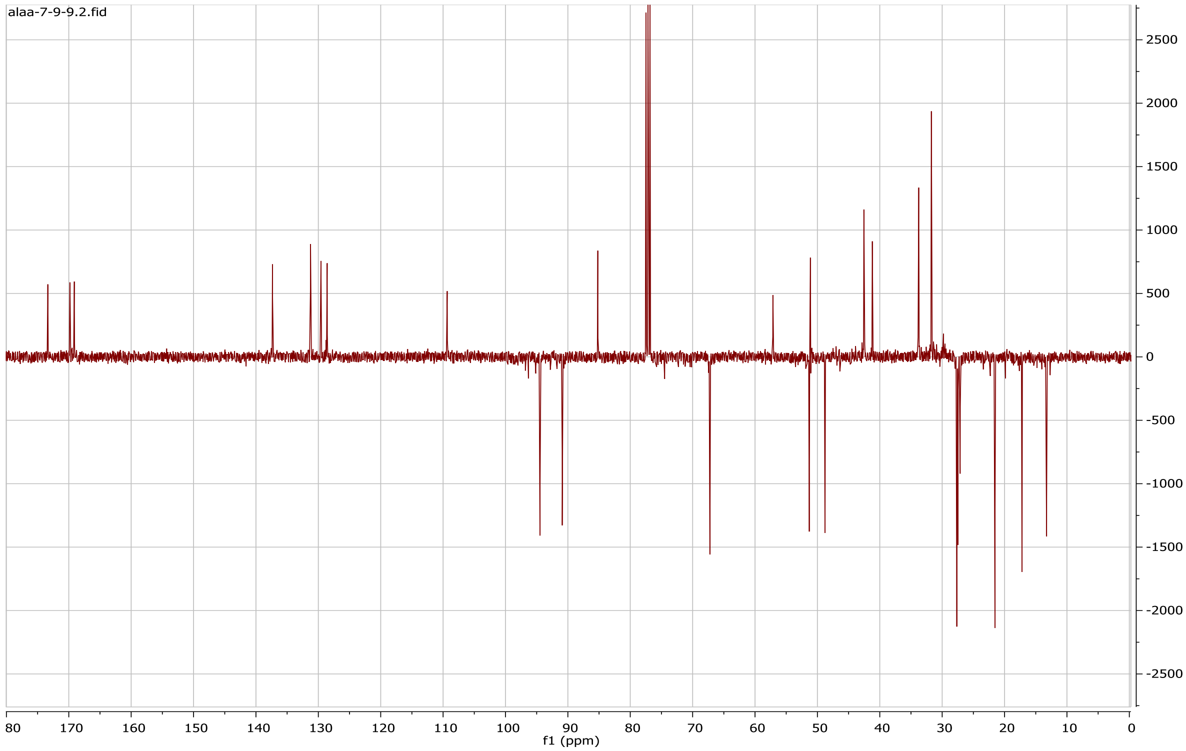

Figure S10. APT NMR spectrum of compound **2** in $CDCl_3$

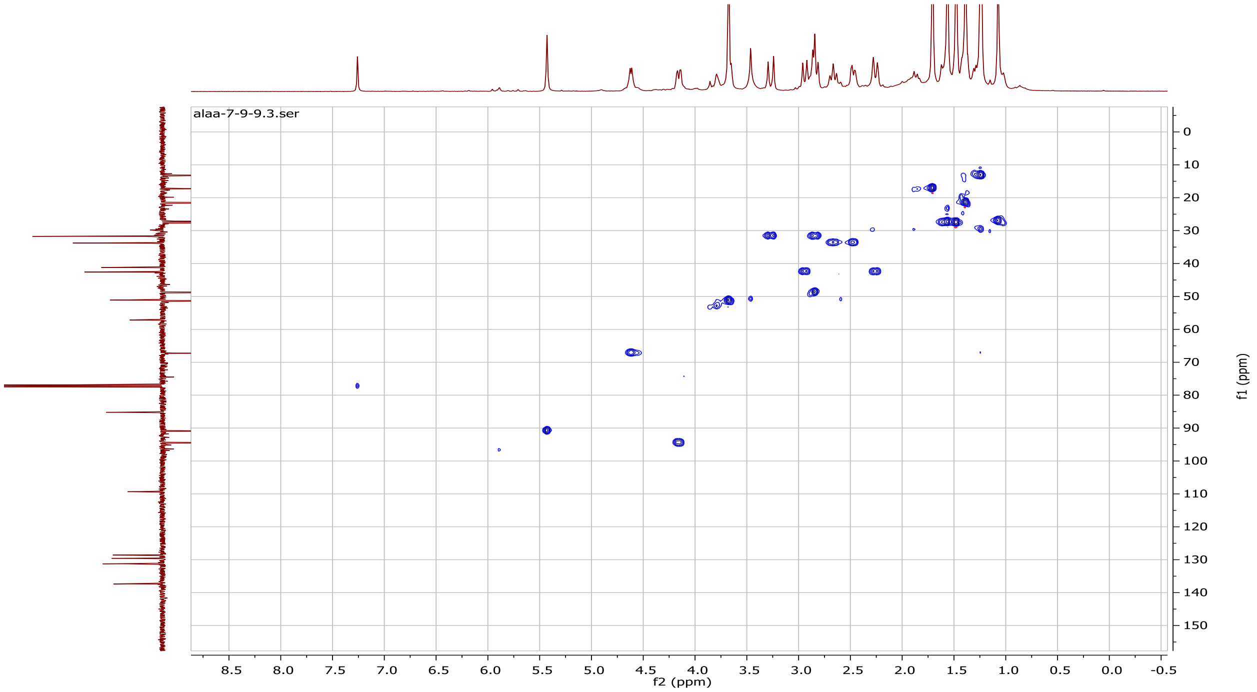

Figure S11. HSQC spectrum of compound **2** in $CDCl_3$

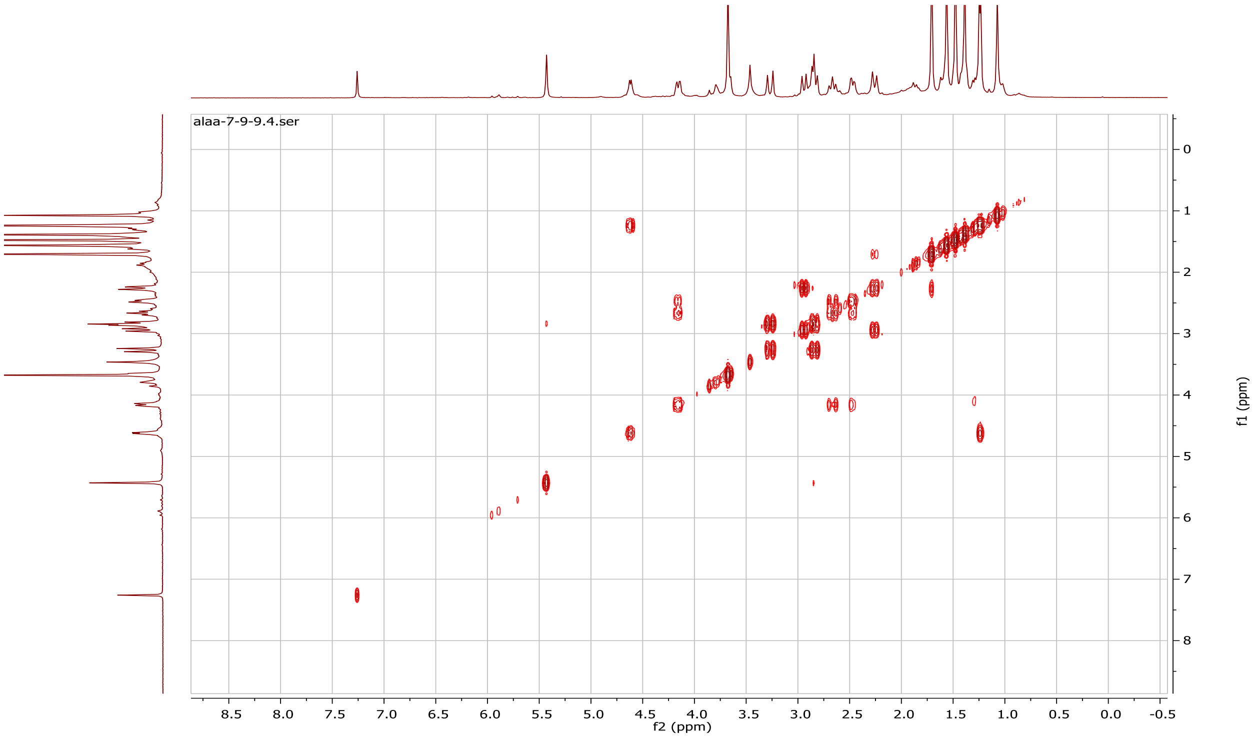

Figure S12. COSY spectrum of compound **2** in $CDCl_3$

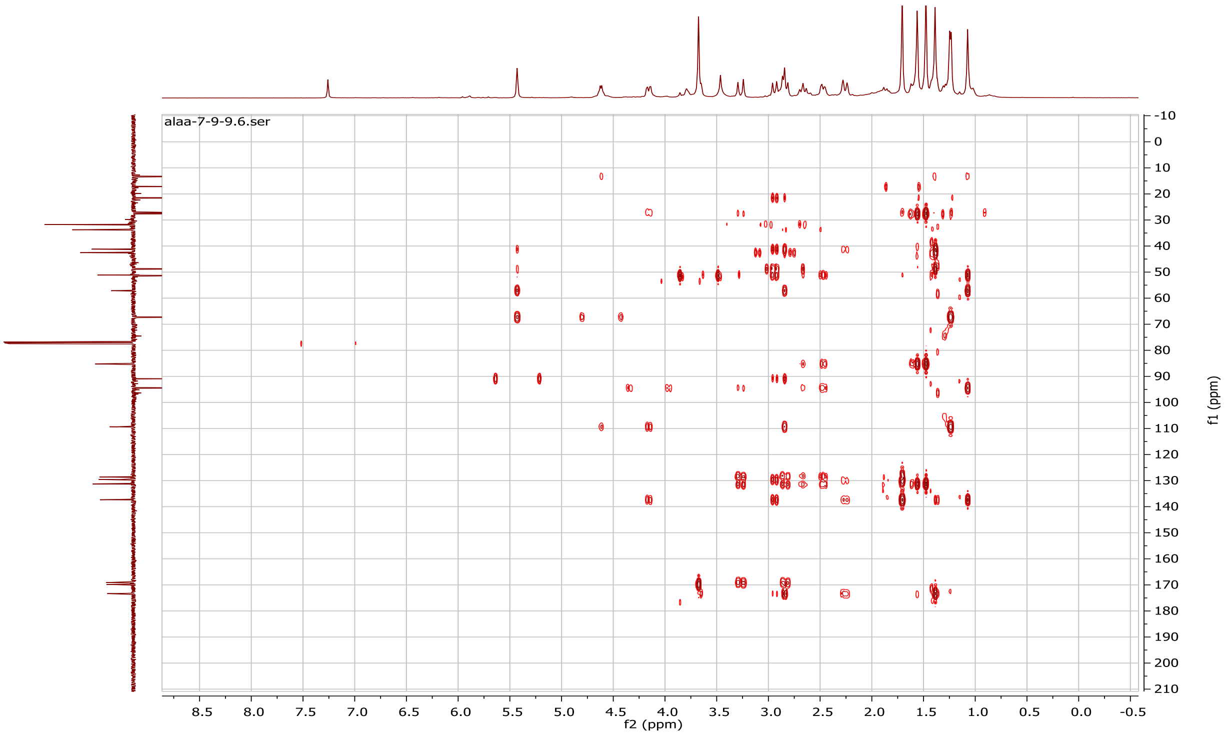

Figure S13. HMBC spectrum of compound **2** in $CDCl_3$

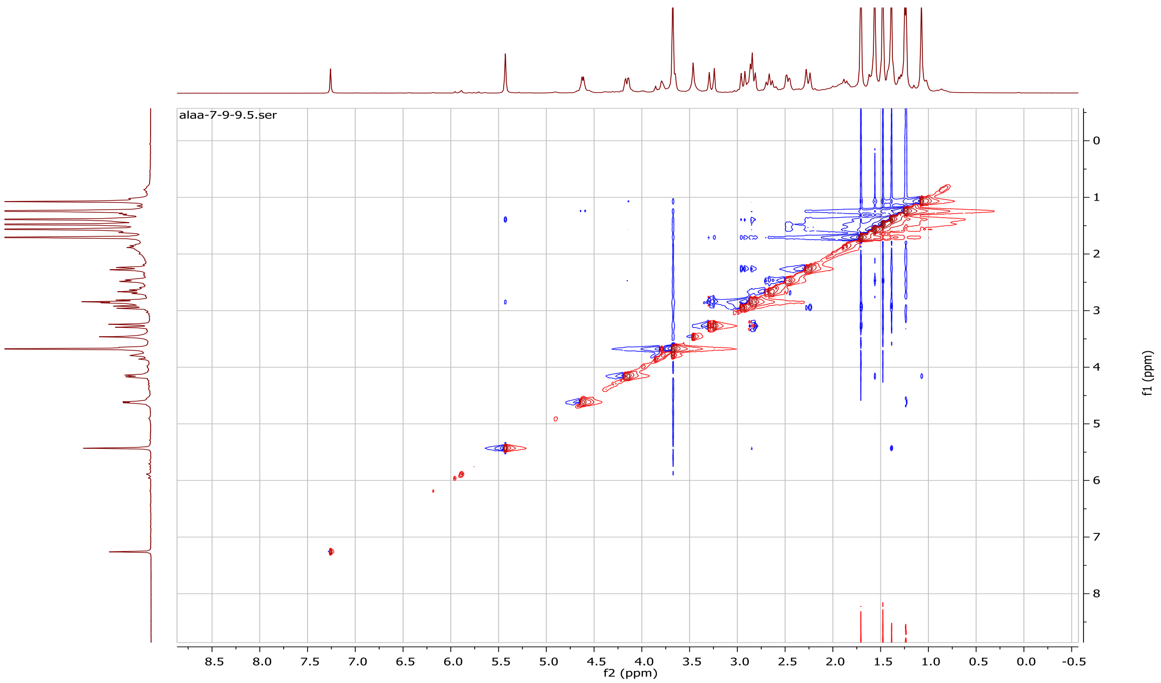

Figure S14. NOESY spectrum of compound **2** in $CDCl_3$

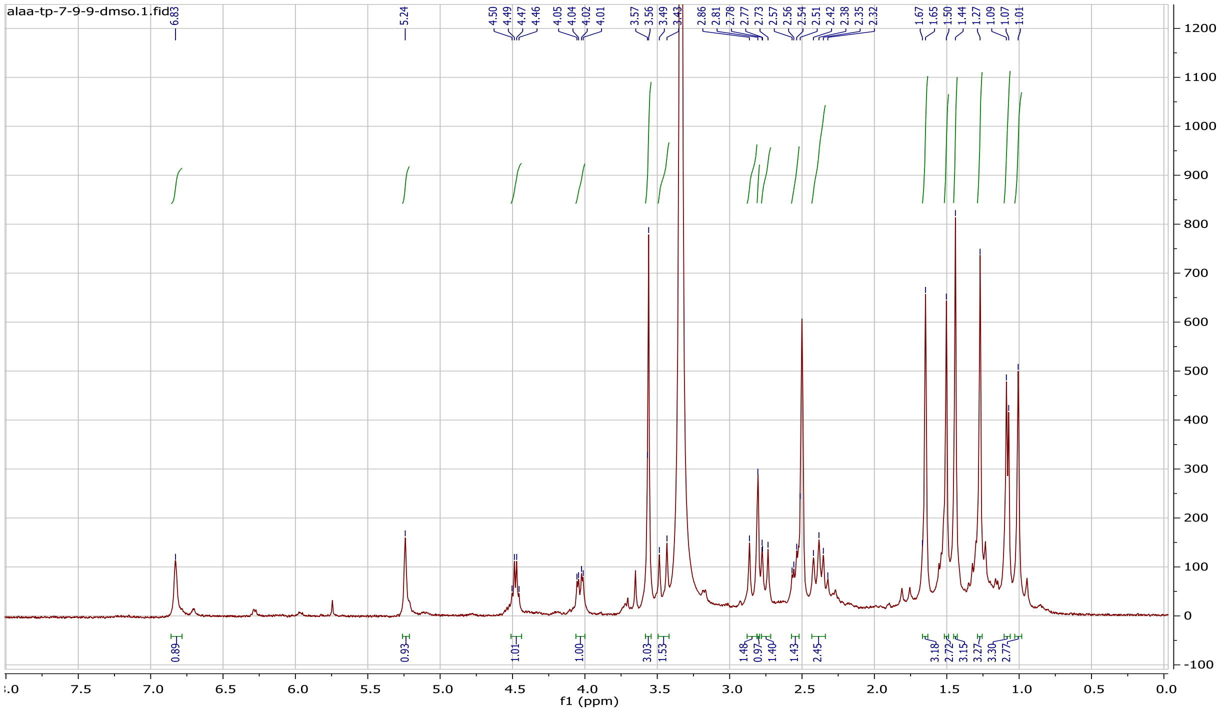

Figure S15. ^{1}H NMR spectrum of compound **2** in DMSO-d_6

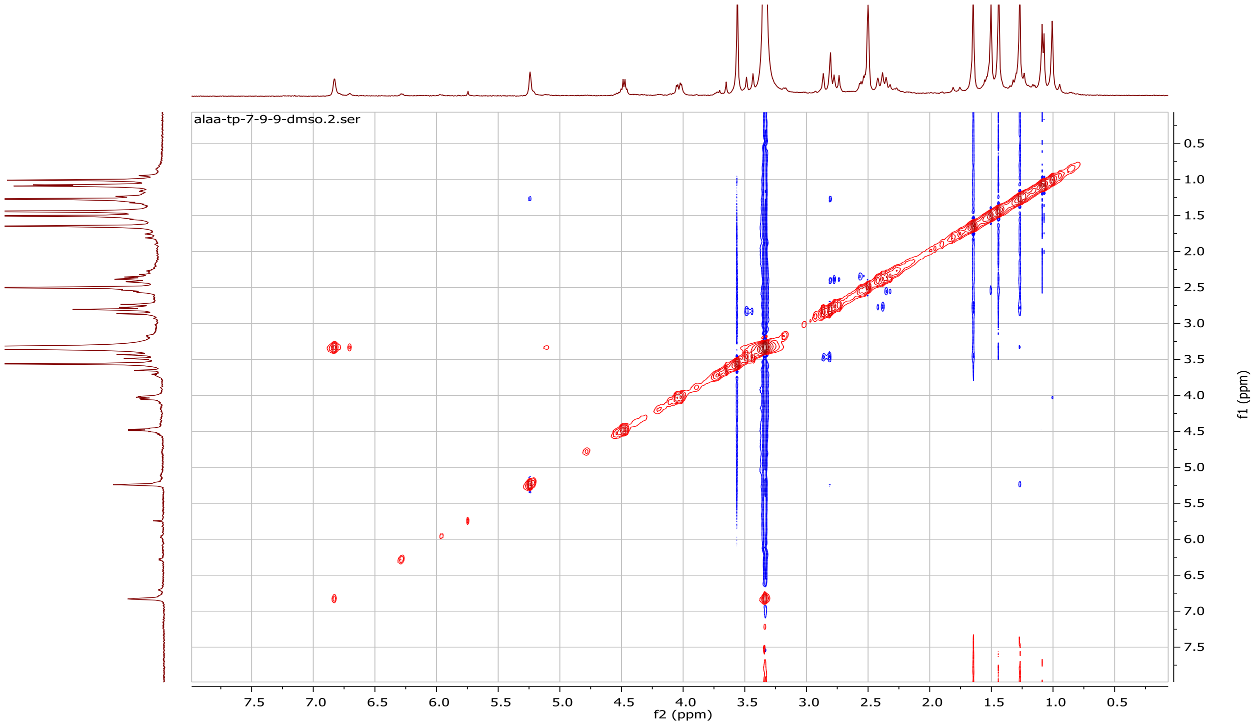

Figure S16. NOESY spectrum of compound **2** in DMSO-d_6
